# An Advanced Energy-Efficient Environmental Monitoring in Precision Agriculture Using LoRa-Based Wireless Sensor Networks

**DOI:** 10.3390/s23146332

**Published:** 2023-07-12

**Authors:** Višnja Križanović, Krešimir Grgić, Josip Spišić, Drago Žagar

**Affiliations:** Faculty of Electrical Engineering, Computer Science and Information Technology, Josip Juraj Strossmayer University of Osijek, 31000 Osijek, Croatia; kresimir.grgic@ferit.hr (K.G.); josip.spisic@ferit.hr (J.S.); drago.zagar@ferit.hr (D.Ž.)

**Keywords:** lora technology, wireless sensor networks, energy efficiency, environmental monitoring, precision agriculture

## Abstract

Sensor networks, as a special subtype of wireless networks, consist of sets of wirelessly connected sensor nodes often placed in hard-to-reach environments. Therefore, it is expected that sensor nodes will not be powered from the power grid. Instead, sensor nodes have their own power sources, the replacement of which is often impractical and requires additional costs, so it is necessary to ensure minimum energy consumption. For that reason, the energy efficiency of wireless sensor networks used for monitoring environmental parameters is essential, especially in remote networking scenarios. In this paper, an overview of the latest research progress on wireless sensor networks based on LoRa was provided. Furthermore, analyses of energy consumption of sensor nodes used in agriculture to observe environmental parameters were conducted using the results of real measurements in the field, as well as simulations carried out based on collected data about real equipment. Optimization methods of energy consumption, in terms of choosing the appropriate data collection processes from the conducted field measurements, as well as the settings of network radio parameters imitating real conditions used in conducted simulations were highlighted. In the analyses, special emphasis was placed on choosing the optimal packet size. Unlike in other papers analyzing energy efficiency of LoRa communication, in this paper, it was proven that the adjustment of the transmission speed to the actual size of the packet is important for better energy efficiency of communication and that it can reduce energy consumption considerably. Moreover, in the paper, the contents of a packet that can be used in precision agriculture is suggested in order to prove that the 6-bit packet is sufficient for energy-efficient collection of parameters from the environment, in contrast to the 11-bit packets used in standard commercially available equipment.

## 1. Introduction

The efficient application of wireless sensor networks in agricultural processes contributes positively to the sustainable development of the economy based on agriculture and food production. The effects of these activities also have a positive impact on the ecology. The processes of monitoring ecological and agronomic parameters that enable a precise assessment of the physiological state of crops enable the timely adjustment of agrotechnical measures to reduce the impact of climate change on agricultural production.

Wireless systems based on the usage of sensor nodes make processes in agriculture more intelligent because they become more precise, data-oriented, and greatly automated [1]. The broad usage of microprocessors and microcontrollers, which include small sensor nodes that can be self-modifiable, low-cost tooling, and scalability, demonstrates that wireless sensor networks can be used in the digitization of agriculture [2]. As they join a large number of sensor nodes into a network, wireless sensor networks represent the base for an extremely heterogeneous IoT environment in which there are many different technologies and communication standards. Such a heterogeneous environment enables optimal adjustment to different areas of sensor application. Every field of sensor application requires a suitable selection of communication systems and parameters to achieve reliable and energy-efficient communication. The aim of the implementation of wireless sensor networks in test environments is to connect sensor nodes, deployed at different remote locations, to servers used to store and process data in a reliable and energy-efficient manner. To enable applied research aimed at increasing the efficiency of implemented wireless sensor networks, the communication processes that occur within the networks are subjected to testing for the selected scenarios. The analyses include the specifics of communication in different scenarios regarding the need for continuous monitoring of network parameters and real-time communication.

The source of energy required for the activities of the sensor nodes is primarily a battery. The sensor nodes can operate from several months up to several years. The battery cycle is affected by numerous different stress factors including temperature, discharge current, charge current, and state of charge ranges. Since the sensor node’s battery life cycle is one of the most severely limited resources in the network, for the implementation of the wireless sensor network especially in areas that are more difficult to access and areas that can be accessed less often, which is a typical example in digital agriculture, it is important to consider the optimization of energy consumption and to achieve the maximum possible energy savings. Therefore, in this paper, the configurations of wireless sensor networks concerning the optimization of the consumption of energy are analyzed. Optimization is analyzed in terms of the choice of adequate star-of-stars topology typical for LoRaWAN networks, data collection, as well as the set-up of network radio parameters. As already confirmed by other sources, for example [3,4], the sizes of the packets sent contribute significantly to energy consumption. Unlike in other studies conducted in this field, for example in [3,4], in this paper, special emphasis is given to finding the optimal packet size with regard to transmission rate in order to find the most energy-efficient size of the packet to be used in precision agriculture. For that reason, unlike other articles, this article gives an example of the contents of a packet that can be used in precision agriculture in order to prove that the 6-bit packet is sufficient for efficient collection of parameters from the environment and higher energy-efficiency of communication, in contrast to the 11-bit packets used in standard commercially available equipment.

The content and contributions of the paper are outlined in the following way: In Section 2, a review of the literature in the field of application of LoRa wireless sensor networks is presented, and the developments in the field carried out in this work are highlighted. In Section 3, the research methodology of issues related to the energy efficiency of LoRa sensor networks is presented, which includes the presented model of energy consumption of the LoRa sensor node, the defined parameters of wireless communication in LoRa networks, the selection of appropriate parameters for LoRa communication, the analysis of aspects of wireless communication based on LoRa with regard to the choice of appropriate volume of traffic in the LoRa network, proposed structure and format of LoRa packets, limitations based on LoRa time-on-air communication, as well as an explanation of unconfirmed and confirmed data transfer processes in the LoRa network. Furthermore, in Section 4, the evaluation and the optimization of energy consumption in LoRa sensor networks are analyzed in detail in selected scenarios, and the results of the collected measurements from the field as well as the results collected based on simulations are presented. The simulations are carried out on the basis of all collected parameters, which were previously shown by field measurements to guarantee energy-efficient communication. Finally, in Section 5, the most important remarks related to the selection of solutions that provide adequate energy efficiency of LoRa-based communication are highlighted.

## 2. Literature Review

The sensors intended to control the cultivation of agricultural crops can monitor environmental parameters such as air temperature, air humidity, soil temperature, soil moisture, chemicals in the soil, leaf wetness, air flow, insolation, luminosity, and solar radiation. The application of IoT solutions is increasingly common in precision agriculture in greenhouses, orchards, and vineyards [5,6,7,8,9]. In order to optimize the parameters of wireless communication, it is necessary to carry out analyses of various communication aspects. Therefore, the optimization of parameters should be carried out to achieve reliable communication with minimal energy consumption, which is the subject of research in this article.

An important criterion in the selection of appropriate technologies is low energy consumption, so that the battery-powered nodes achieve a sufficiently long autonomy and multi-year life cycle expectancy. Therefore, technologies from the group of Low Power Wide Area Network (LPWAN) technologies, high-range low-power wireless technologies, are suitable for application in precision agriculture scenarios [10]. The specifics of LPWAN technologies are a long communication range for sending information over greater distances and low power consumption. These features make LPWAN technologies very practical for open-area IoT implementations. There are many LPWAN technologies and new ones are constantly emerging and improving, but currently, LoRa, Sigfox, and Narrowband IoT (NB-IoT) are leaders in the field [11,12]. The advantage of the application of these technologies in networking relates to the fact that the end devices working with these technologies are designed to consume energy efficiently and minimally [13]. Low power and narrow bandwidth allow for very low power consumption used in operation [14].

Its low power associated with long-range communication makes LoRa the most prominent among LPWAN technologies [15]. Of all LPWAN technologies, LoRa has probably involved consideration of most scientific studies concerning problems, current solutions, and open issues [13,16,17,18,19,20,21,22,23,24,25,26,27,28,29,30,31,32,33,34,35,36,37,38]. A significant number of surveys has been conducted with the aim of analyzing requirements, deployments, and challenges of LoRa technology [13,16,17,18,19,20,21,22,23,24,37,38]. Therefore, available surveys [13,16,17,18,19,20,21,22,23,24,37], from 2019–2023 are listed and briefly described hereafter. In [39], a systematic literature review of published studies on LoRa applications was conducted using articles from 2010–2019. The study [16] presents an overview of LoRa networking, covering the technological difficulties in setting up LoRa infrastructures, such as connection management, allocation of resources, consistent communications, and security. In [17], a comprehensive survey of LoRa from a systematic perspective is given. It includes analysis of LoRa communication processes, security, and its enabled applications. In [18], the landscape of available simulators for IoT and wireless sensor networks is explored and their performance is compared. Moreover, LoRa networking performance metrics, including range, throughput, energy usage, and security is studied in [13,19]. In [20], the literature survey presented in the paper describes LoRa performance under different scenarios and a few implementation obstacles of this technology. Furthermore, in the papers [21,22], a discussion of the advantages of LoRa over the existing technologies used in IoT is presented. Also, in [23,24], a detailed description of the LoRa technology is given with regard to existing security and reliability mechanisms. Also, in [38], a survey of Machine Learning techniques for wireless sensor networks is presented. Finally, in [37] a review of WSN-based agricultural applications was presented and a comparison was conducted among different wireless technologies or protocols, such as Wi-Fi, Bluetooth, ZigBee, GPRS/3G/4G, LoRa, and SigFox. None of these surveys considered the methods of energy efficiency of LoRa implementations in precision agriculture based on the amount of network traffic and the size of sent packets with regard to transmission rate, which is the subject of research in this article.

The majority of the research conducted on LoRa and LoRaWAN has been focused on features such as coverage, range, network capacity, throughput, delay, scalability, and robustness, for instance in [25,26,27,28,29,30,40,41,42]. However, limited consideration of features such as energy consumption has been given, unlike the considerations in this article. It is essential because many LoRa devices are not powered by the grid.

Prompted by the challenges of energy consumption, some recent works have focused on the power dissipation of sensors within wireless sensor networks [15]. Although the analyses in the majority of the proposed papers suggested to estimate the amount of energy consumed by a sensor node, LoRa technology was not directly included in their energy models [43]. Thus, for instance, in [44], an energy model for ultra-low power consumption of sensor nodes was presented. However, the radio frequency module used in the study did not include LoRa technology. Another model of energy estimation was presented in [45] aimed to achieve low energy consumption of sensor nodes. In order to save energy, it was concluded that the microcontroller and the communication module must be idle as long as possible when they are not active. Although these papers provide useful results, LoRa technology was not considered in the performed analysis, as is the case in this article.

Furthermore, some other studies which took LoRa communication into account did not study the optimization of the consumption of energy of LoRa sensor nodes. For instance, in [46], a comparison of LoRaWAN classes was presented and their energy consumption models were proposed. Although the obtained results are based on real measurements, the paper did not study the effects of LoRa parameters such as communication range, coding speed, and transmission power level on the total energy consumed, which is the subject of research in this article. The analyses presented in [31] deal with this topic to a limited measure, providing only incomplete estimates on parameters related to LoRaWAN energy efficiency. However, they did not consider the practical behavior of the hardware of the LoRaWAN device, nor the effect of the main LoRaWAN mechanism settings and parameters, which is also the subject of research in this article.

Moreover, only a few works provided data relevant to the current consumption of LoRa and LoRaWAN devices, obtained from a manual or by experimental measurements [31,32,33,34,36,47]. Such details correspond to device states of sleep, transmission, as well as reception [47]. Although the developed models enable the characterization of lifetime and energy costs of LoRaWAN device, these models do not include, for instance, energy consumption used within the processing state of sensor units, which is also the subject of research in this article.

In addition, based on the information relevant to transmit, receive, and sleep states of a LoRa/LoRaWAN device, a few analytical models of LoRa and LoRaWAN energy consumption have been published, for example, in [31,36]. They include the lifetime of sensor nodes and the energy cost of data delivery. However, these models are also very basic since consideration needs to be given to several other states for a LoRa and LoRaWAN device that exist and are involved in communication. These include, for instance, a detailed calculation of time for message transmission, provided only in [48]. It is important to note that the aforementioned study only examines LoRa, and therefore does not model the use of acknowledgments and retransmission which are important mechanisms of the MAC layer defined in LoRaWAN, which is set to be the subject of research in this article.

Although some studies show the energy consumption of sensor nodes based on both LoRa and LoRaWAN communication, the presented values are obtained from a data table or empirically [49,50,51,52,53,54,55]. However, the studies in question did not consider an energy model that can thoroughly estimate and optimize the energy consumption of sensor nodes, which is also the subject of research in this article.

Considering the results of the research studies provided so far, as well as the aforementioned deficiencies in those studies, in this article a detailed analytical model of energy consumption of sensor nodes and energy cost of data delivery is provided. The model considers the hardware behavior and mechanisms of real LoRaWAN devices, as well as their physical and MAC layer parameters. The proposed energy consumption model is based on Class A, as the most energy-efficient LoRaWAN class. Based on the literature review, it can be concluded that this paper is among the first to specify a detailed analytical model of energy consumption and simulation of the energy cost of data delivery of sensor nodes. It considers both the real hardware behavior of LoRaWAN devices and real-case tests of data packet delivery in different specific fields of LoRa deployment scenarios. While the actual hardware behavior of LoRaWAN devices includes, for instance, their levels of power consumption in different modes of operation, the real-case tests of data packet delivery conducted in different specific LoRa field deployment scenarios enable decision making on whether a packet delivery confirmation is required in a particular application scenario or not. The defined energy model is supplemented by the optimization of LoRaWAN parameters important for the reduction in energy consumption of sensor nodes, such as spreading factor, coding rate, and bandwidth, as well as the generated traffic volume. It is necessary to emphasize that the impact of LoRaWAN parameters such as payload size, acknowledged transmission, coding rate, spreading factor, and communication range on the sensor node consumption is also studied in [15]. In contrast to the analyses in [15], in this paper, special emphasis is placed on the analysis of optimization of energy consumption with regard to packet transmission rate considering different sizes of data packets which are not all very common in commercial devices, as well as energy consumption aspects in different scenarios of LoRa network deployment.

Furthermore, unlike the analyses carried out in [52], in which the battery lifespan is calculated without the use of acknowledgments, this paper presents the possibility of using acknowledgments to estimate the lifespan of batteries more precisely. Furthermore, the impact of deficiency of data delivery caused by degradation on the energy performance of LoRaWAN was considered. For actual analysis, the simulated models are derived based on measurements conducted in the real testbeds. The possibility of using acknowledgments or omitting them is also analyzed, based on the results collected by real measurements, in order to select the best case for the optimization of energy consumption. In addition, the models used in this paper also study the impact of collisions on LoRaWAN energy performance.

Moreover, unlike the analyses in [53], in which the focus is placed on the optimization of the fixed LoRaWAN setup communication with only a single considered data rate, this paper analyzes the use of different available data rates to select the best method for the optimization of energy consumption. The model considers the possibility of the use of an adaptive data transmission rate, as presented by results of conducted simulations.

Finally, unlike the analyses conducted in [3,4], which considered large-scale LoRa network deployments with up to 100 densely distributed sensor nodes, in this paper, analyses are conducted for precision agriculture application scenarios in which deployments of up to 20 optimally distributed sensor nodes are sufficient for the monitoring of environmental parameters. In addition, unlike the energy profile used in [3,4], the selected energy profile used in the simulation process is based on the estimated energy consumption of a transceiver [54] due to its energy efficiency and application in the conducted field measurements. All the procedures for modeling the energy aspects of LoRa communication processes applied in the model presented within this paper enable a more precise assessment of the optimal combinations of parameters to achieve a reduction in the energy consumption in the selected specific use cases, whereby a special emphasis is placed on the analysis results of the optimization of energy aspects related to defining the optimal packet size with regard to traffic rate.

## 3. Methodology

### 3.1. Energy Consumption Model of Lora Sensor Node

The energy consumption modeling process represents an essential consideration when designing sensor nodes to monitor a specific application. For most applications, such as the ones presented in [55], the communicating sensors should perform the following: sense events, process the local information of sensed events, and transmit packets to the access point [56]. Each task consumes power over a given period. Therefore, an accurate energy consumption model of the sensor node is essential to estimate the sensor lifetime, as proven in [15,57]. Furthermore, the energy model allows the power consumption optimization of the sensor node.

In order to study the sensor node autonomy, it is necessary to model the different operating modes of the sensor node. Then, the consumed energy of each mode can be determined, and the consumption energy model can be defined.

**Figure 1:**
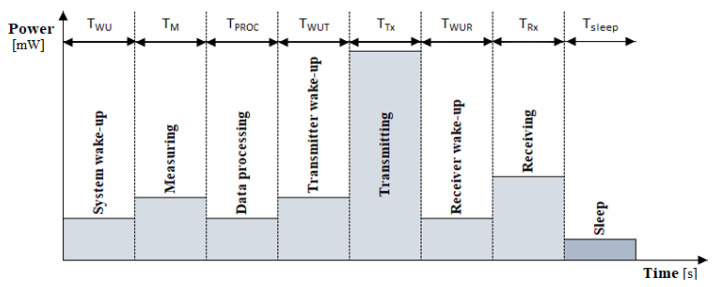
Energy consumption states of the sensor node.

Most of the time, the sensor node is put into sleep mode. That is a low-power mode, but the energy consumed in this mode can also impact the total energy consumption of the sensor node. The total consumed energy E__Total_ of the sensor node for one cycle is:

E__Total_ = E__Active_ + E__Sleep,_ (1)

where E__Sleep_ and E__Active_ represent the dissipated energy by the sensor node in the sleep mode and the total energy consumption during the active mode of the node microcontroller, respectively [15]. The duration of the sleep mode should be ≥99%, and the duration of the active mode should be ≤1% [15]. In addition, E__Sleep_ is expressed as:

E__Sleep_ = P__Sleep_ · T__Sleep,_ (2)

where P__Sleep_ and T__Sleep_ are the power consumption and the time duration in the sleep mode, respectively [15]. The total energy consumption in active mode E__Active_ is calculated as the sum of the energy consumption used for the operation of each part of the sensor node. Therefore, it is given by the following equation [15]:

E__Active_ = E__WU_ + E__M_ + E__PROC_ + E__WUT_ + E__Tx_ + E__WUR_ + E__Rx,_ (3)

where the consumed energies that should be considered are the ones for the system wake-up, data measurement, microcontroller processing, the wake-up of the transmitter, the transceiver’s transmission, the wake-up of the receiver, and the reception of the transceiver, respectively [15], as presented in Figure 1.

Energy consumption in specific operating modes can be determined regarding the current consumption of LoRa/LoRaWAN transceiver modules, as declared in the specifications and affirmed by conducted measurements, presented in Table 1. As can be seen, the sleep current ranges from 0.1 μA to 34 mA. Sleep current for the hardware platforms is up to several orders of magnitude greater than sleep current of their transceivers [58], which can be near 1 μA, or even lower than that [47]. In addition, the power consumption can be determined from the transceivers’ electrical specifications under the stated supply voltage conditions, as presented in Table 2.

The output power is sensitive to the power supply voltage, and their performance is typically expressed at 3.3 V [64]. For instance, the HopeRF RFM95/96/97/98(W), used in the field measurements whose presentation is given in Section 4 of the paper, has a minimum output supply voltage of 2.4. V and a maximum of 3.7 V, which applies to the high-power +20 dBm operation [54]. According to [54], the following can be quoted:

(a) Transmit mode of operation of the transceiver

In transmit mode, the power consumption, presented in Table 2, is optimized only when packet data needs to be transmitted by enabling the following blocks: RF, PLL (used for calibration of the receiver), and PA (amplifier capable of yielding RF power) [64]. The transceivers feature three different RF power amplifiers [54]. Two of these, connected to RFO_LF and RFO_HF, can deliver up to +14 dBm and are not regulated for high power efficiency [65]. The third, PA, connected to the PA_BOOST pin, can deliver up to +20 dBm via a dedicated matching network [58]. The low-frequency PA_LF covers the lower bands (up to 525 MHz), while the high-frequency PA_HF covers the upper bands (from 860 MHz). Finally, the high-power PA_HP, connected to the PA_BOOST pin, covers all frequency bands the transceiver addresses.

(b) Listen mode of operation of the transceiver

In this mode, the circuit spends most of the time in an idle state, during which only the RC oscillator is on. Then, periodically, the receiver wakes up and looks for an incoming signal. If a signal is detected, the receiver is kept on, and the data are analyzed. Otherwise, if there is no signal for a defined period of time, the receiver is switched off until the following receive period [64]. The radio stays mainly in a low-power mode during the listen mode. This fact results in very low average power consumption.

(c) Receive mode of operation of the transceiver

The receiver automatically restarts after a packet collision or after receiving a valid packet. In receive mode, the transceiver can detect packet collisions and restart the receiver. Collisions are detected by a sudden rise in received signal strength. This functionality can be useful in network configurations where many asynchronous slave nodes attempt periodic communication with a single master node [64]. Depending on the application and the environment, there are various ways to implement listen mode: wake on a Preamble Detect interrupt, wake on a SyncAddress interrupt, or wake on a Payload Ready interrupt [64].

### 3.2. Energy Profile of LoRa Sensor Nodes

Each sensor node is characterized by an energy profile and LoRa parameters. The node’s behavior is designed as specified by [70,71,72,73], as presented in Table 3. The energy consumption model based on LoRa, which estimates the consumed power of different states of sensor nodes, is used. The default energy profile used in the simulation process, whose presentation is given in Section 4 of the paper, is based on the energy consumption of [54], due to its energy efficiency and application in conducted field measurements. Therefore, the energy consumption values of the selected transceiver listed in the specifications [54] are used. However, the simulations are not solely constrained to a single energy profile, since a comparison was made of the selected transceiver with other transceivers referenced in the literature relevant to the research area. The power consumption optimization in the selected scenarios is conducted by adopting specific power reduction settings of communication parameters. For instance, a reduction in the power consumption of the radio frequency (RF) mode is applied. As a result, it minimizes the total power consumption of sensor nodes since RF modules (for Tx and Rx) use considerable power.

During the simulation process, different energy profiles are allocated to nodes, imitating various nodes, whereby the average values of energy consumption in different states are used. The different energy states of the simulated nodes are summarized in Table 3 according to sources in the cited literature and the technical report of the transceiver used in the conducted field measurements. A similar experiment has been conducted in [3], but there are differences between the energy profile in transmit and receive modes. As can be observed from Table 3, the power states that are considered are transmit, receive, sleep, and processing, as well as waking up and setting up the radio.

The simulation process assumes that all nodes have an initial transmit power of 14 dBm, which conforms to the LoRaWAN specification. This power level, which corresponds to the low-power operation mode, enables lower energy consumption than the high-power operation mode, but also sufficient signal coverage of the area, which was previously proven by field measurements. Moreover, only the operations in the channels required by the LoRaWAN specification are considered, i.e., 868.1 MHz, 868.3 MHz, and 868.5 MHz.

As already proven, optimizing the LoRa parameters such as SF, CR, and payload size are crucial in reducing the sensor node’s consumed energy.

A spreading factor SF12 should be used in the second receive window [70]. However, in this work, a confirmed message was sent with SF10. A lower spreading factor results in a faster reception, yielding lower energy consumption at the node. The spreading factor SF10 is favored since the areas in the selected scenarios are well covered by the LoRa signal, which can be concluded according to the previously presented field measurements [52]. Moreover, a lower spreading factor results in faster data reception, which causes a lower node energy consumption. However, since the LoRa gateway handles a downlink channel by allowing for a higher duty cycle and higher transmit power operation, a loss of packets due to lowering the spreading factor can be resolved by transmitting data at higher power, as also stated in [74].

Therefore, within the simulation process, it is assumed that the downlink message scheduled for the first receive window (Rx1) uses the same frequency and data rate as the uplink messages. In the second receive window (Rx2), a fixed predefined frequency and data rate (for SF10) are used.

### 3.3. Selection of Adequate Parameters for LoRa-Based Communication

The LoRa-based access networks implemented in open spaces connect sensor nodes in different remote locations to centrally located storage and data-processing servers. The range of communication is an essential parameter when considering the effectiveness of implemented solutions regarding the implementation of sensor networks in remote areas to monitor environmental and agronomic parameters. In most cases, the high data transfer rate is not so significant. However, it is necessary to ensure the energy-efficient transmission of data over relatively long distances. The parameters described hereafter affect LoRa transmission range and energy efficiency [54].

LoRa operates in the sub-GHz unlicensed frequency bands. The LoRa systems operate at the ISM frequency bands, and the carrier frequency (CF), the center frequency used for the transmission band, is 863 MHz to 870 MHz in Europe.

Moreover, there are also some regulatory constraints in most countries on the permissible occupied bandwidth (BW) [54]. Increasing the signal bandwidth (BW) allows a higher effective data rate to be used, thereby reducing the transmission time at the expense of reduced sensitivity of the receiver. It is possible to choose between 125 kHz, 250 kHz, or 500 kHz. However, in the multi-data-rate channel mode, the gateway is limited to a 125 kHz bandwidth with typically eight channels, even though the maximum bandwidth of LoRa is 500 kHz. Therefore, if there is a need for a high data transfer rate, the best choice is the value of 500 kHz. On the other hand, if it is necessary to achieve a long communication range, it is necessary to choose the bandwidth value of 125 kHz. The greater the bandwidth, the shorter the time-on-air value, and the sensitivity of the receiver is better. However, a higher time-on-air value will also mean higher energy requirements.

The spreading factor and error correction rate allow the optimization of the trade-off between occupied bandwidth, data rate, link budget improvement, and immunity to interference.

The spread spectrum LoRa modulation is performed by representing each bit of payload information by multiple chirps of information. A chirp is a sinusoidal signal whose frequency monotonically increases (upchirp) or decreases (downchirp) [60]. A chirp is a pulse that sweeps from f_Low_(−BW/2) to f_High_(+BW/2) [60]. The number of chirps forms a symbol. For instance, if a symbol value is between 1 and 128, the number would be one of the combinations of 2^7^ = 128 chirps. The Spreading Factor (SF) represents the number of chirps per symbol. Its value is an integer number between 6 and 12. The values of SF from 6 to 12 represent 64, 128, 256, 512, 1024, 2048, and 4096 chirps/symbol, respectively. The greater the SF value, the more ability the receiver has to remove the noise from the signal. Thus, the greater the SF value, the more time is taken to send a packet, but a higher range will also be achieved because the receiver’s sensitivity is better. For example, if the expansion factor is minimal, i.e., SF = 6, a higher speed can be achieved, but with a reduction in the possible range. SF = 6 presents a particular use case for the highest data-rate transmission possible with the LoRa modem. The spreading factor must be known in advance on both transmit and receive sides of the link, as different spreading factors are orthogonal to each other. Generally, according to the LoRaWAN specifications, the spreading factor control is limited to 6 unique spreading factors going from spreading factor 12, or the lowest data rate, to spreading factor 7, for the highest data rate. This means up to six sensor nodes can transmit data simultaneously on the same channel.

Due to interference, some of the data bits can be lost, so the error correction bits are used to recover the original lost data bits. LoRa uses Forward Error Correction (FEC) techniques to increase the robustness of radio communication links. FEC requires error correction bits, redundant bits, to be added to the data. Thus, error coding incurs a transmission overhead. Although FEC reduces the data throughput, it increases the receiver’s sensitivity. LoRa defined a set of values which are referred to as Code ∈ {1, 2, 3, 4} to calculate the Coding Rate (CR) based on the following equation [67]:

(4) $$CR=\frac{4}{4+\mathrm{Code}}$$

Hence, CR = {4/5, 4/6, 4/7, 4/8}, and it denotes that every four useful bits are encoded by 5, 6, 7, or 8 transmission bits, while the overhead ratio is 1.25, 1.5, 1.75, and 2, respectively. The coding rate could maximize the data rate if fewer code bits are used. The smaller the coding rate is, the higher the time on air is to transmit data, but the prolonged data transfer time will also affect the battery consumption.

The packet header can be optionally included in the coding rate for use by the receiver. However, if more redundant bits are sent to the receiver, LoRa will consume more power. Therefore, in response to channel conditions, the coding rate and the robustness to interference can be changed.

In addition, the relationship among LoRa transmission parameters is also relevant for energy consumption. If the bandwidth of LoRa is constant, the chirp rate differs according to the SF [67]:

(5) $$R_{c}=BW\cdot R_{s}=\frac{BW^{2}}{2^{SF}}\;\left[\mathrm{chirps}/s\right]$$

For each SF, the orthogonality of the chirp prevents interference with any other [62]. Thus, when two or more transmitters use the same channel to transmit data with different SFs simultaneously, the packets do not interfere with each other [68]. With knowledge of the key LoRa parameters, bandwidth, and the spreading factor, the LoRa symbol rate can be controlled [54]:

(6) $$R_{s}=\frac{BW}{2^{SF}}\;\left[\mathrm{symbols}/s\right]$$

(7) $$SF={log}_{2}(\frac{R_{c}}{R_{s}})$$

Moreover, depending on the SF in use, LoRa bit rate ranges [54]:

(8) $$R_{b}=SF\cdot \frac{\left[\frac{4}{4+CR}\right]}{\left[\frac{2^{SF}}{BW}\right]}\cdot 1000\;\left[b/s\right]$$

Data rate (DR) can also be expressed considering LoRa transmission parameters [54]:

(9) $$DR=SF\cdot \frac{BW}{2^{SF}}\cdot CR\;\left[b/s\right]$$

The higher the SF and the lower the channel bandwidth, the lower the data rate is and, thus, the longer the data transmission time is [68].

The analysis of the effect of different values of SF and CR LoRa radio parameters on the consumed energy and range is performed in [69]. According to the presented results, the following can be concluded. When the SF value is high, the time necessary for data transmission increases. This means that the sensor node consumes more power to transmit data. The reason for setting a high SF value is to maximize the transmission range as one of the most critical factors in field deployment, as also proved in [15]. Furthermore, when the coding rate increases, the data transmission time and the consumed energy decrease. Therefore, when a long range needs to be ensured, energy consumption can be regulated by the CR factor. By increasing the CR value from 4/8 to 4/5, the energy consumption can be reduced while maintaining the maximal range enabled by the high SF parameter value [69]. However, this depends on the radio conditions because it could imply more retransmissions which would cause more energy consumption.

### 3.4. Choosing the Adequate Volume of Traffic in the LoRa Network

The aim of analyzing the impact of the amount of transmitted network traffic within the network on the energy consumption for one-hop star topology scenarios is analyzed in [69], as quoted within this chapter. First, the scenario where all nodes send data at the same time interval, a typical case in rural scenarios for sending data collected from the field, is analyzed. It is concluded that node placement in a star topology presents a vital determinant of sensor node lifetime. However, although adequate signal coverage of the area must be achieved, the nodes should be as close as possible to the gateway to reduce energy consumption. Moreover, regarding the energy depletion of individual nodes, it is concluded that the star topology is more suitable for application to sensor networks than the cluster topology, in which the energy of cluster head (CH) nodes is depleted the fastest. Since CH nodes are difficult to replace, this causes the loss of functionality of the entire cluster, while in the star topology, the depletion of energy of an individual node does not cause such a loss in data delivery from the area it covers with its signal. In addition, it is concluded that in scenarios where sending a more considerable amount of data is unnecessary, as in the case of monitoring the state of yield, energy savings should be achieved by sending an adequately determined smaller volume of data from sensors. Finally, it is concluded that significantly less energy consumption can be noted within the same time interval when sending data with higher delay, i.e., less frequently. Thus, from the presented results, applying LoRa technology is an adequate solution in IoT scenarios where fast transmission of large amounts of data is not required, as in precision agriculture. In precision agriculture, additional energy savings can be achieved by sending smaller data sets over extended periods between transmissions. Considering these conclusions, optimizing energy consumption in a LoRa network requires consideration of the optimal packet size. To save energy, sending as little total traffic as possible is necessary. Therefore, optimizing packets to maximize information transfer in the minimum number of bits is vital [75]. Sending the highest value information, with the right balance of redundancy, compactness, and timeliness, should provide the best results in applying a sensor network based on LoRaWAN for monitoring environmental and agronomic data essential for precision agriculture.

The LoRaWAN specification, defined for each worldwide region, provides for a maximum data payload size N. This maximum payload size varies by data rate (DR) because of the maximum on-air transmission time allowed for each regional specification. For example, for EU863-870, the maximum payload size for DR0-DR2 is 51 bytes, for DR3 it is 115 bytes, for DR4-DR7 it is 222 bytes, while for DR8-DR15 it is not defined [75]. If DR limits are restricted only to higher values, for instance, never below the chosen DR, this also limits how far away signals can be received, so this should be considered when choosing a packet length. Moreover, while 51 bytes in the payload in Europe may not be very restrictive, in the regions that operate in the 900 MHz ISM band, the lowest DR is restricted to 11 bytes [75]. However, for all DRs other than DR0, the available maximum packet size is equal to or greater than the EU863-870 region. Therefore, if the same packet format should be used in all regions, and DR0 is chosen to be supported (since a lower DR typically means a more extended range), the maximum payload length for any packet would be 11 bytes. If the sensor node is restricted to operate above the DR0, the maximum payload becomes 51 bytes.

Since the sensors can monitor parameters related to both the environment and the state of the crops, the data in the packet payload is used to transmit measured values, for example, temperature, humidity, pressure, solar radiation, and rain. When sending data packets, it is crucial to maximize the transfer of useful information. Hence, it must be considered that the payload contents are used to maximize the transfer of useful information with minimal transmission bandwidth. Therefore, a critical aspect of data transmission planning refers to the processes for determining the necessary update rate of information to make a minimum number of samples. A reasonable sampling rate should be used to capture the original data. Moreover, for highly periodic measurements, as is the case for monitoring of most parameters related to precision agriculture, an adequate sampling rate will result in an accurate representation of the measured values. Thus, more frequent data delivery is necessary for monitoring data related to the amount of precipitation essential for irrigation procedures, especially in the rapid occurrence of short-term storms. In these cases, it is necessary to take several samples per hour to assess the current state as accurately as possible.

It is logical to group all readings into the same packet in the case of sensors with multiple sensor elements that have synchronized measurements. In the case of group readings, bits are often arranged in registers of a fixed size. However, in this case, there is an unnecessary waste of resources allocated for unused bits. For example, some sensors have 10- or 12-bit resolution, while LoRaWAN provides slots of 8 bits (or multiples of 8) for transmission, where it is possible that some of these bits remain unused. Therefore, optimizing the data transfer between devices is necessary to maximize the transfer of useful information in the minimum number of bytes.

In a group reading, the process of maximizing data transfer in the fewest bits, i.e., the process of bit packing, takes advantage of binary payload data transmission where payload bits are sent one after the other to ensure a compact flow of information. An example of such a message format as can be found at the commercially available all-in-one sensor weather stations is presented in Table 4. The values presented in Table 4 represent the collected data and contents of the actual packet with a payload of 11 bytes.

However, an essential aspect of bit packing is ensuring the appropriate scale and precision of the data. Many physical measurements can be compressed into smaller data representations, so sending more bits than required for the same data is inefficient.

For example, for transferring information about environmental temperature, an 8-bit representation can be used instead of the 11-bit. It enables the representation of a temperature range from −40 °C to +87.5 °C, sufficient to display the required temperatures with high accuracy. The same applies to other environmental parameters monitored by sensors, such as the relative humidity sensor, which can measure environmental humidity or soil moisture at different depths, and the pressure sensor or rain gauge which is used to determine the maximum instantaneous rainfall rate. Irrigation planning based on monitoring current conditions in the environment carried out by sensor nodes represents a relevant example of the increasingly necessary application of sensor networks in precision agriculture, especially regarding the necessity of optimal water utilization, which is essential in the face of increasingly frequent water shortages. The proposed 6-byte payload format shown in Table 5 corresponds to the optimal data set for monitoring environmental parameters essential for the crop irrigation process, which provides the same amount of useful information as the previously shown 11-byte payload, but within the reduced packet size.

### 3.5. Time on Air Constraints of Communication Based on LoRa

To optimize the overall energy consumption in the network, LoRaWAN facilitates the control of the air time and data rate [3]. To effectively manage the regulatory constraints of time on air and receiver sensitivity, it is necessary to be able to calculate the total on-the-air transmission time of a LoRa packet for a given configuration of communication parameters, i.e., spreading factor (SF), coding rate (CR) and signal bandwidth (BW), as defined hereafter. For calculating the time on air, it is convenient to define the symbol duration T_symb_. The symbol duration of a LoRa symbol is defined as [76]:

(10) $$T_{symb}=\frac{2^{SF}}{BW},$$

This is the time taken to send chirps at the chirp rate, recalling that the bandwidth defines the chirp rate. The symbol duration is based on the spreading factor (SF) and the bandwidth (BW). Each LoRa symbol comprises 2^SF^ chirps, each covering the entire bandwidth.

Every LoRa packet consists of a preamble and data. The preamble contains only upchirps, while the data part comprises upchirps with discontinuities (the position of the discontinuities in frequency is what encodes the transmitted information). Therefore, LoRa packet duration can be calculated as the sum of the duration of the preamble and the transmitted packet. The preamble length is calculated as follows [54]:

(11) $$T_{preamble}=(n_{preamble}+4.25)T_{symb},$$

where n_preamble_ is the number of programmed preamble symbols.

The payload duration depends upon the header mode enabled, implicit (headerless) or explicit (with header) modes. The number of symbols that make up the packet payload and header are given by [54]:

(12) $$payloadSymbNb=8+\max(ceil\left(\frac{8PL-4SF+28+16-20H}{4\left(SF-2DE\right)}\right)\left(CR+4\right),0),$$

with the following dependencies: the number of payload bytes PL, the spreading factor SF, the header (H = 0 when header is enabled, and H = 1 when no header is present), the data rate optimization (DE = 1 when the low data rate optimization is enabled, DE = 0 when it is disabled), and the coding rate CR (from 1 to 4).

If the time on air requires reduction, and the packet length is known in advance, then the header information can be removed once the destination of the packet is known. The payload duration is then the symbol period multiplied by the number of payload symbols [54].

(13) $$T_{payload}=payloadSymbNb\cdot Tsymb,$$

Then, the time-on-air, or packet duration, is simply the sum of the preamble and payload duration [54]:

(14) $$T_{packet}=T_{preamble}+T_{payload},$$

LoRaWAN devices must comply with the regulations imposed in the industrial, scientific, and medical (ISM) radio bands in which they operate [3]. These regulations include a limitation in the duty cycle of transmission and excited transmit power [77]. The duty cycle indicates the fraction of time a resource is busy. LoRaWAN enforces a per-band duty cycle limitation. In Europe, duty cycles are regulated by Section 7.2.3 of the ETSI EN300.220 standard.

Since every radio device must comply with the regulated duty cycle limits, this applies to sensor nodes and gateways. For example, ETSI regulations limit the duty cycle over one-hour intervals, whereas LoRaWAN enforces compliance with such limitations over the interval between transmitting a message and the next one. Moreover, on The Things Network’s public community network, a Fair Use Policy applies, which limits the uplink air time to 30 s per day per node and the downlink messages to 10 messages per day per node. If private networks are used, these limits do not apply, but the governmental and LoRaWAN limits apply.

To stay within these limits, the easiest way to do this is to calculate how much air time each packet consumes to choose a good transmit interval. For instance, as presented in Table 6, the calculator [48] is used for the chosen packet payload size (the maximum of which is 51 for low data rate (SF12), up to about 222 bytes for the best conditions (SF7)), the chosen packet header size (which is at least 13 bytes for the header for regular uplinks, or a total of 23 bytes for a Join Request), the chosen coding rate (from 4/5 to 4/8, where higher values mean more overhead), the chosen number of preamble symbols (which is 8 for all regions but can be different using plain LoRa),the chosen bandwidth (which is typically 125 kHz, but can be 250 kHz or 500 kHz), the chosen spreading factor (where “higher” means more range and better reception, but also more air time), and the chosen total air time to send the entire packet (in ms). Therefore, as presented in Table 6, the calculated values include the TTN Fair Access Policy-based number of average messages/day and average messages/hour when sending all day, as well as the duty-cycle-based time between the start of the next packet in the same sub-band. ETSI regulations limit the duty cycle to one-hour intervals so as not to crash its servers by generating excessive traffic, and not to directly improve energy savings.

As can be seen from the conducted calculations presented in Table 6, by complying with the TTN Fair Access Policy, since more messages/hour can be sent for smaller packets, for a maximum packet size of 51 bytes, a total of 10 packets/hour cannot be exceeded. The control of the air time is achieved by adapting the data rate, i.e., by changing SF values. Also, the values are presented for the three chosen values of packet application payload sizes. As the presented values show, the decrease in the spreading factor results in a lower air time. However, a higher air time allows the receiver to demodulate the message better.

Instead of defining a specific data rate, LoRaWAN specifies the data rate as a combination of a spreading factor and bandwidth [47,78]. It can be seen that DR3 uses SF9, while DR2 uses SF10. Hence, with DR3, a double amount of data can be sent simultaneously, compared to DR2 [4]. Similarly, since the symbol time for DR3 is half the symbol time for DR2, there will also be half the energy per bit if the same transmit power is used. Likewise, this is also true for SNR [4].

### 3.6. Battery Lifespan

Choosing the optimum SF parameter, which enables the highest possible transmission speed, leads to a longer battery life [48].

Despite the better range, a sensor node will consume more energy when transmitting with a higher spreading factor [3]. Therefore, in addition to modifying the spreading factor value, the transmission power can be altered to increase the range further or decrease the energy consumption.

Considering the limitation in the duty cycle, after transmitting a message, every sensor node needs to wait Toff seconds before transmitting data again in that band (periodicity) [54]:

(15) $$T_{off}=\frac{T_{air}}{T_{dc}}-T_{air},$$

In the case of sending a message with a payload size of 51 bytes and a spreading factor of 12 while respecting a duty cycle limit of 1% (which dictates that the time for data transmit/hour is 36 s), the time off is 4 min and 57 s.

Since the LoRa gateway utilizes a downlink channel allowing for a higher duty cycle and higher transmit power operation, a loss due to lowering the spreading factor can be resolved by transmitting at a higher power [4]. However, an optimal combination of energy consumption regarding limited battery life should be achieved.

To achieve a longer sensor node battery lifetime, e.g., in the order of years, the LoRaWAN nodes need batteries with greater capacity than typical button cell batteries, e.g., of AA type, but these are more expensive [47]. The results presented in [47] show that an appropriately configured LoRaWAN sensor node powered by a battery of 2400 mAh can achieve a 1-year lifetime if sending a message every 5 min and a theoretical lifetime of 6 years for infrequent communication. Moreover, an energy consumption of 0.05–0.44 mJ and a battery lifetime between one and 13 years, respectively, are obtained for a device running on two AA batteries when transferring data from 1 to 10 times per hour [53].

For the chosen packet payload sizes, the calculator [79] is applied to estimate the differences in energy consumption for several types of batteries which can supply sensor nodes, as presented in Table 7. The assumptions considered are processing power of 15 mW for 5 ms, confirmed reading of a sensor value per each period, and sleep mode consumption of 10 µW. The worst-case results have been calculated for the usage of SF12, transmission power of +14 dBm, and with the acknowledgment in the second Rx window. The best-case results have been calculated for the usage of SF7, transmission power of +2 dBm, and with the acknowledgment in the first Rx window. As can be seen from the comparison of the collected results, a substantial energy consumption reduction can be achieved by transmitting smaller data packets.

Since sensor nodes are supplied with cell batteries upon delivery, purchasing a solar panel initially represents an additional cost in contrast to the sensor nodes, which are only battery-powered. The cost of a set of cell batteries represents approximately 12 times the cost of a cheaper sensor or approximately 2 times the cost of a more expensive sensor [80]. Additionally, the cost of a solar panel represents 8 times the cost of a cheaper sensor, or 4/3 times the cost of a more expensive sensor. For instance, while temperature and luminosity sensors are cheaper, humidity and atmospheric pressure sensors are examples of more expensive sensors.

In the case when the duration of the battery’s lifetime is short and frequent battery changes are required, the application of solar power supply represents an effective solution in the long term. However, due to the limited capacity and lifetime of batteries, they need to be replaced after a particular time, even if solar power is used.

However, in sensor networks where more sensor nodes are implemented, the energy consumption per sensor node for the exact total generated network traffic in the same period becomes lower than when fewer sensor nodes are implemented. Thereby, the battery life per each sensor node is extended, and the battery needs to be changed less often, which demands less energy from the solar collector. In Table 7 data related to battery consumption are presented in cases where different packet sizes, different numbers of messages, and different network parameters, such as SF, are used. As presented, the longest battery life is achieved when smaller packets are sent, when packets are sent less often, and when SF7 is used, and not SF12.

## 4. Results and Discussion

### 4.1. Evaluation of Communication Range and Channel Attenuation Modeling

Different scenarios are selected to analyze, in actual conditions, the propagation of LoRa signals at different distances and in different environments. The propagation of LoRa signals in rural environments (with and without obstructions such as a forest), suburban environments, and urban environments are considered. Two stations, transmitting and receiving, are examined, providing point-to-area and point-to-point communication services. The environments in which line-of-sight is impossible due to obstructions are taken into consideration in the conducted analyses. Some realistic case study scenarios are considered, in which the distance between the receiver and the transmitter is less than 100 m, 500 m, 2 km, 5 km, and 7 km, respectively. Therefore, the chosen distances between the receiver and the transmitter are much lower than the theoretical and experimental range described in the literature [81], which refers to open environments under line-of-sight (LOS) conditions. In the rural LOS scenario, the transmitter antenna is placed above a grove of trees. In contrast, in the considered NLOS scenarios, the transmitter antenna is positioned so that the signal is partially propagated through trees or the signal propagation is partially obstructed by buildings. In the analyzed cases conducted in this research, the gateway is placed at the height of approximately 65 m above the ground.

The field experiments are conducted using hardware prototypes based on LoRa devices to determine the state and range of communication links. In real testbeds, measurements are made using an RFM95/96/97/98(W) LoRa module to determine how parameters such as transceiver antenna gain, spreading factor, and coding rate influence communication. Different parameters are used to measure signal strength and signal-to-noise ratio at various distances between the transmitter and receiver and to determine whether it is possible to achieve reliable communication in different areas, and under what conditions.

The conducted study considers operation in the European region, in the EU863–870 ISM band, where three default channels are defined: 868.10, 868.30, and 868.50 MHz. Each of these channels has a bandwidth of 125 kHz, uses LoRa modulation, and must allow data rates from 0.3 kbps to 5 kbps (DR0 to DR5), depending on the SF value set.

Considering the compromise between range and interference immunity, and energy consumption, two transmission power modes, a low-power (LP) mode and a high-power (HP) mode, are compared, and their impact on the communication range is analyzed. The LP mode with the transmission power of +14 dBm considers high efficiency and low current consumption (25 mW). The HP mode with the transmission power of +20 dBm considers a more extended range and robustness but has a higher current consumption (100 mW). The duty cycle of transmission at +20 dBm is limited to 1%. Although the transceiver supports transmit power of up to +20 dBm, according to frequency regulations, such power is permitted only for one frequency channel. However, any of the six channels can use +14 dBm.

The LoRaWAN protocol is used for long-distance, low-power communication. A common problem that occurs in for instance, remote rural areas, as well as in some other specific deployments, such as in forests [82], is the lack of a seamless power source that allows the installation of gateways near an area that needs to be covered by the LoRa signal. Also, this causes the occasionally complex interconnection of gateways with the infrastructure of the core network. In these cases, it is necessary to use a more extended communication range. While communication over long distances of multiple kilometers is used for IoT, the narrow bandwidth can make it unreliable, and there may be significant losses in the delivery of sent packages. Moreover, since the line of sight is not always free of obstacles from vegetation or buildings, this imposes NLOS conditions that impair transmission quality.

**Figure 2:**
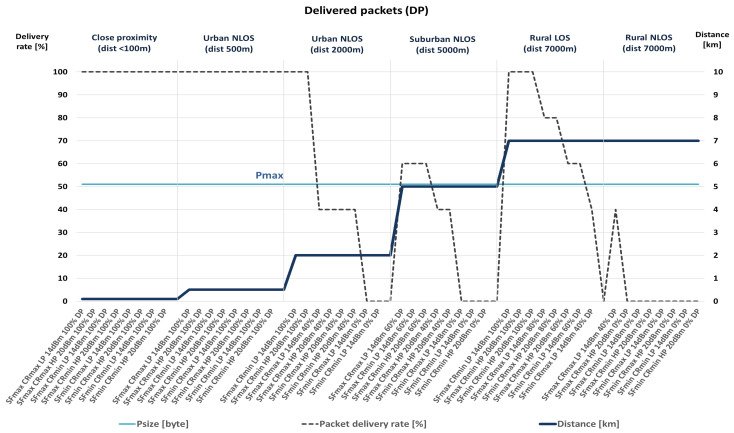
Delivery rates of packets with the maximal size.

**Figure 3:**
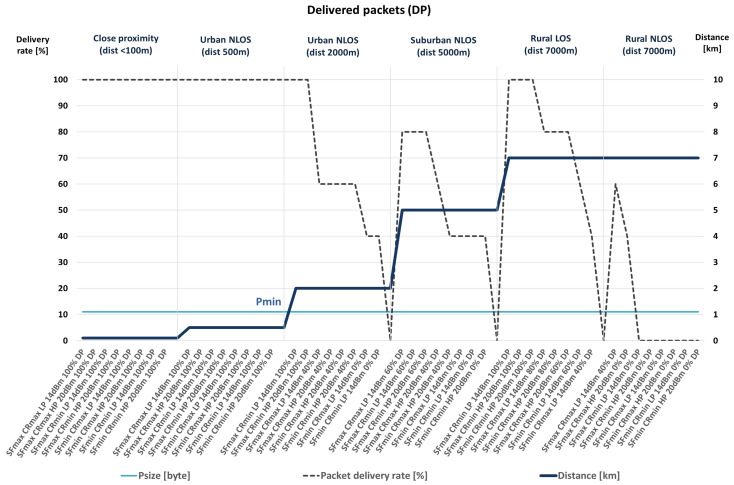
Delivery rates of packets with a minimum size.

The performance metric used is packet delivery percentage. Therefore, the conclusions about the transmission quality, according to measured packet delivery percentage, and the combinations of communication parameters that give the best results are presented hereafter, as well as in Figure 2 and Figure 3.

As presented within the NLOS scenarios, some unsuccessful transmissions of packets are noted. The number of unsuccessful transmissions is more significant with a greater distance and less visibility between the transmitter and the receiver.

During the measurement and data-collection process, the LoRa signal’s bandwidth was set to 125 kHz. Moreover, the transceiver is configured to use different values of spreading factor, two of which are selected for more detailed analyses. These are SF = 7 (minimum value) and SF = 10 (optimal energy consumption if a more extended range needs to be ensured). Furthermore, the LP mode with the transmission power of +14 dBm for low current consumption (25 mW) and the HP mode with the transmission power of +20 dBm with higher current consumption (100 mW) are chosen. Table 8 shows the amount of delivered packets compared to packets sent with different configurations of SF and CR network parameters in different scenarios. Accordingly, 100% indicates that all sent data has been received. Circled values are those in which the most efficient data transfer was achieved in each of the scenarios, considering the combination of network parameters that represents a more energy efficient solution.

The presented results indicate that, among all considered scenarios, the most critical packet deliveries are in remote suburban and rural areas with no direct optical visibility since NLOS conditions impair transmission quality. Therefore, data with the best values for a particular scenario were selected among the obtained data. In doing so, the differences in the obtained results should be considered with regard to the selected packet sizes. The selected packet size values were Pmax = 51 bytes and Pmin = 11 bytes.

As shown in Table 8, during the scenario selection process, the selected scenarios (the ones with the white background) are those in which the percentage of successful transmissions is the highest among all collected results within each particular scenario. However, when several options have the same percentage of packet delivery, the combinations of communication parameters that enable lower energy consumption are chosen within the selection process. According to previously conducted analyses, they include combinations in which smaller transceiver power values are selected (LP mode, compared to HP mode) due to lower energy consumption, a lower value of the SF parameter (7, compared to 10), if possible, considering the quality of communication (the higher percentage of packet delivery), and the higher value of the CR parameter (4/5).

The presented results reveal that there is no packet loss up to a distance of 500 m, regardless of the NLOS conditions. Moreover, within 2 km and at a more extended range from the transmitter, in the case of NLOS (non-line-of-sight blocked by some obstacles and possible interference from other radio systems), there are situations where a complete loss of communication occurs. However, the number of delivered packets can be increased with adequately selected communication parameters, as, for instance, higher SF and, if necessary, lower CR values since combinations with minimum CR are more susceptible to noise. Moreover, the following must be observed. Despite some sources, the packet delivery level increases with the amount of data sent. Also, with a shorter packet length, the percentage of packet delivery is higher in some cases. Therefore, even in NLOS conditions at distances over 5 km, it is possible to achieve communication, while it is necessary to consider the usage of higher SF values.

In further analyses of the quality of communication, the performance metrics used on the receiving end are the Received Signal Strength Indicator (RSSI) and the Signal-to-Noise Ratio (SNR). The conducted analyses consider the power received at the end of the symmetrical half-duplex communication link between the transmitter and the receiver.

Measurements of received signal strength (RSSI) and noise impact on the signal (SNR) enabled conclusions about the transmission quality concerning the achieved packet delivery rate, as presented hereafter, with the highlighted combinations of communication parameters that produce the best results.

The RSSI measures the incoming signal power at the RF input port, evaluated within the receiver bandwidth. This value is absolute in units of dBm and with a resolution of 0.5 dB. The RSSI value is usually used to determine the most significant possible distance between the transmitter and the receiver since the signal strength decreases as the distance between the transmitter and the receiver increases [52]. However, the signal strength also depends on the interference, i.e., the noise level. Therefore, the RSSI can sometimes indicate incorrect positioning of equipment [83]. In order to minimize the effects of obstacles on communication between the receiver and transmitter as much as possible, during all measurements, the gateway was placed at a height slightly above 60 m. Nevertheless, even then, direct visibility between the sensor node and the gateway was not enabled because the sensor node, considering its purpose, was placed too low to receive the most valuable results of the measurement.

Moreover, the value of signal versus noise represents the comparison of the signal strength to the noise power. It is denoted as SNR, and its unit of expression is dB. SNR is the best indicator of obstacles in signal transmission. A positive SNR value means the signal is stronger than the noise, and a negative SNR means that the noise overpowers the signal [84].

For the listed combinations of communication parameter settings, measurements of RSSI and SNR values are carried out using the RFM95/96/97/98(W) LoRa module. For the signal strength at shorter distances, higher RSSI values are obtained. The obtained RSSI values are around −80 dBm at distances less than 100 m, at distances of 500 m between −100 and −110 dBm, and at greater distances from −110 to −130 dBm.

Moreover, with increasing distance between the transmitter and the receiver, the values of the SNR parameter decrease. In NLOS scenarios, the influence of obstacles on the SNR levels can be noted as well. The SNR values become negative at distances ≥2 km, which means that the received signal operates below the noise floor.

In addition, the moderate success of the collected transmissions within each scenario is calculated for the measured range of RSSI and SNR values, and some unsuccessful transmissions are noted. Finally, as presented, the number of lost packets is recorded for the RSSI parameter values less than −110 dBm and the negative SNR parameter values.

**Figure 4:**
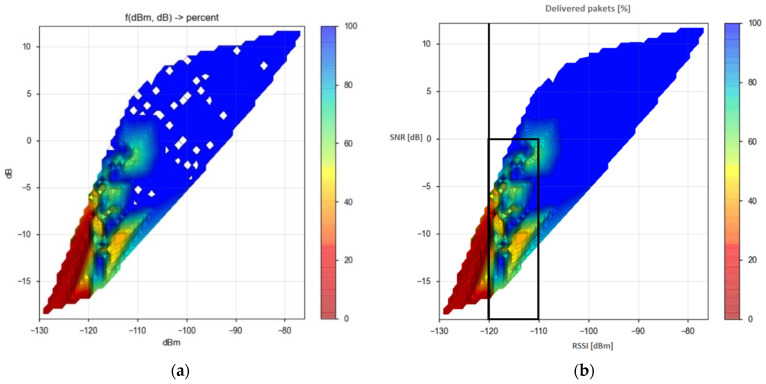
Limits of reliable data transmission. (**a**) Original picture after conducted measurements; (**b**) Retouched version of original picture with the displayed rectangle within which confirmations of packet delivery are required, and black line on the left of which packets have not been delivered.

According to the specifications of the transceiver used in the measurements [54], under an extended range mode of operation, and in the case of 125 kHz bandwidth, the following RF sensitivity levels can be realized: −118 dBm for SF = 6, −123 dBm for SF = 7, −126 dBm for SF = 8, −129 dBm for SF = 9, −132 dBm for SF = 10, −133 dBm for SF = 11, and −136 dBm for SF = 12. A message is received if the signal strength is higher than the receiver’s sensitivity. During the measurements, the LoRa signal’s bandwidth was set to 125 kHz, and the transceiver was configured to use the different values of spreading factors. As presented in Figure 4, obtained from the analysis of the collected results, this enabled receiver sensitivity of up to approximately −130 dBm.

**Figure 5:**
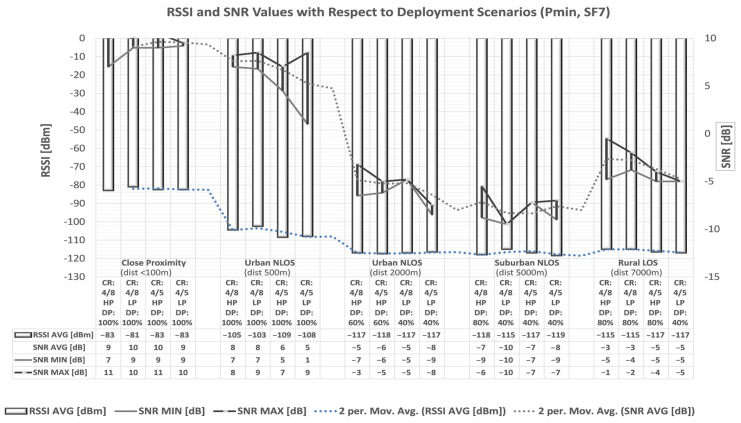
Comparison of measured RSSI and SNR values.

The results presented in Figure 4 and Figure 5 were obtained from approximately five hundred conducted measurements of SNR and RSSI values, as described in more detail in [52]. Several spots with missing values were found in Figure 4, evident after analyzing the collected data from Figure 5 and from all additional sets of SNR and RSSI values with SF7-SF10 [52]. The spots were then filled and values were calibrated. Therefore, Figure 4 was updated from scratch and presented in this form after the retouching process to obtain a complete view of the values. According to the collected results about the unsuccessful transmissions, it can be concluded that for SNR[dB] < 0, for the purpose of a reliable data transmission, confirmations, i.e., acknowledgments, are also necessary. The results of the conducted research in this paper are first collected for all measurements in all scenarios to obtain Figure 5 presenting the limits of reliable data transmission. Based on it, it is possible to check whether the measurement results obtained in real time fit into the areas where an acknowledgment of packet delivery is required or not. If the measurement data is within the framed area (i.e., if values of RSSI are between −120 dBm and −110 dBm), there is a higher probability that a packet delivery confirmation is required and it can be sent. For RSSI values lower than −120 dBm (i.e., if values are between −130 dBm and −120 dBm) the loss of packets occurs and confirmation of its delivery is necessary.

The values of SNR at which transmission errors, i.e., BER, occur within the communication process depend on the value of the SF parameter and, to some extent, on the CR values as well [76,85,86]. To quantify this, it is assumed that all losses are caused by channel gain variability [86].

To reduce data transmission losses in LoRa communication, it is possible to use the Adaptive Data Rate (ADR) mechanism for optimizing data rates, air time, and energy consumption in the network. The ADR mechanism controls a sensor node’s spreading factor, bandwidth, and transmission power to optimize device power consumption while ensuring that messages are still received at the gateways.

Generally, the sensor nodes close to gateways should use a lower spreading factor and a higher data rate, while sensor nodes further away should use a high spreading factor because they need a higher link budget. When ADR is in use, the network server is capable of increasing the data rate and can indicate to the sensor node that it should reduce its transmission power, while the node can use an automatic data rate reduction when it detects that it is not receiving any response from the network. It is important to note that ADR should be enabled only if a sensor node has stable RF conditions.

The network server takes the 20 most recent uplinks as the input for the optimal data rate, starting when the ADR bit is set. These measurements contain the frame counter, SNR, and the number of gateways that received each uplink. The maximum SNR of the last 20 received uplink messages is used to determine the quality of the link between the node and gateway. If the SNR is lower than required (e.g., if BW = 125 kHz is considered, for SF = 6: SNR = −5 dB, for SF = 7 (DR5): SNR = −7.5 dB, for SF = 8 (DR4): SNR = −10 dB, for SF = 9 (DR3): SNR = −12.5 dB, for SF = 10 (DR2): SNR = −15.5 dB, for SF = 11 (DR1): SNR = −17.5 dB, and for SF = 12 (DR0): SNR = −20 dB), the network server tries to decrease the data rate or increase the transmit power [4].

According to the obtained results of the conducted measurements shown in Figure 4, it can be seen that the area where increased packet losses occur due to signal weakening is located precisely in the area of SNR values specified as marginal for the application of ADR. The most significant losses occur at SNR values lower than −5 dB if the RSSI values are lower than −110 dBm. Furthermore, it is also evident that, in the case when the SNR values are lower than −5 dB and the RSSI values are in the range from −110 dBm to −120 dBm, data delivery still occurs, while in the case when the RSSI values are lower than −120 dBm, the result is a complete packet loss.

### 4.2. Evaluation and Optimization of Energy Consumption in Simulated LoRa Networks

Considering everything stated so far, a prime focus of the conducted research in the following part of the paper is on a detailed analysis of the impact of payload size on energy consumption based on simulation process conducted using simulator [87]. Input values used in the simulation are the ones presented in Section 3.2 describing energy profile of LoRa sensor node, as well as all parameters presented in Section 3 and Section 4.1. The selected payload sizes correspond to real-life applications related to monitoring environmental parameters used in precision agriculture. Therefore, the maximum possible payload size of 51 bytes, an average size of 11 bytes (used in most commercially available applications), and a minimum size of 6 bytes (proven optimal for the chosen precision agriculture application) are considered.

Thus, the analyses are conducted to quantify the impact of relevant LoRaWAN parameters and mechanisms on energy consumption in the scenarios in which different packet payload sizes are used.

In order to save energy, non-time-critical data can be accumulated. Furthermore, by increasing the payload size, the overhead related to header information decreases, and the number of retransmissions in a stable propagation environment reduces. For this reason, it was assumed that the grouped measurements of different parameters are sent within the same packet for the analyses conducted in this paper.

Typical applications of sensor networks in precision agriculture for environmental monitoring are time-related. Collecting current data is essential to obtain more detailed information about specific agrometeorological parameters, for example, the amount of precipitation, such as rain, in specific, more frequent periods. Hence, it is vital to provide a considerable amount of helpful information.

As opposed to the default transmission rate (λ) of 0.02 bits per second, equivalent to transmitting a 9-byte message every chosen hour, for instance, in [3], the data transmission rate is adjusted to the quantity of data that should be transmitted, i.e., to the actual packet size, as well as the packet transmitting interval. The interval is set to 10 min. This means the packet is transmitted every 10 min, so six packets are transmitted per hour. Moreover, the overhead data are considered for assessing the accurate transmission rate, as presented in Table 9. Unlike the previously conducted analyses [3,4], in which the transmission rate does not match the size of the packet and is set to a fixed rate of 0.19 × 10^−3^, this approach considers the actual packet size in determining the transmission rate. This is the main difference between the previously conducted analyses in [3,4], and the analyses conducted in this work, which result in more precise results of the subsequent simulation procedures.

One of the possible energy efficiency mechanisms is reducing the total volume of sent data and an adaptive data sampling process. With less frequent data transmission, the overhead of starting and initializing a transmission is also lower. As already proven, approaches based on reducing the number of packets transmitted can improve the success rate of packet delivery. According to the TTN Fair Access Policy, for a maximum packet size of 51 bytes, a total of 10 packets/hour cannot be exceeded. Therefore, the transmission interval is set to 10 min, which means that data is transmitted every 10 min, so six packets are transmitted per hour. Although the sampling period of 10 min is more extended than the minimum of approximately 5 min, determined by considering duty cycle regulations, it is still short when energy savings are considered. However, it is coordinated with the necessary data related to measuring the amount of precipitation, especially in the case of short-term intense precipitation, after which the need for irrigation can be significantly reduced. The network’s capacity is reduced, not only due to transmissions in the downlink but also due to the off-period time following these transmissions.

### 4.3. Overview of Simulation Results

The performance of the network and individual sensor nodes is evaluated based on the number of uniquely received packets and the mean ratio of the number of uniquely received packets on the gateway to the uniquely transmitted packets per node, i.e., the data extraction rate. This indicates how reliably the intended payload bytes are received by the gateway. Moreover, this differs from the packet delivery success ratio because it does not include retransmissions in the calculation. Hence, the main objective is that the intended payload is received by the gateway, while the number of retransmissions necessary to achieve this goal is of secondary importance. The collected results of the simulation process are presented and compared hereafter.

**Figure 6:**
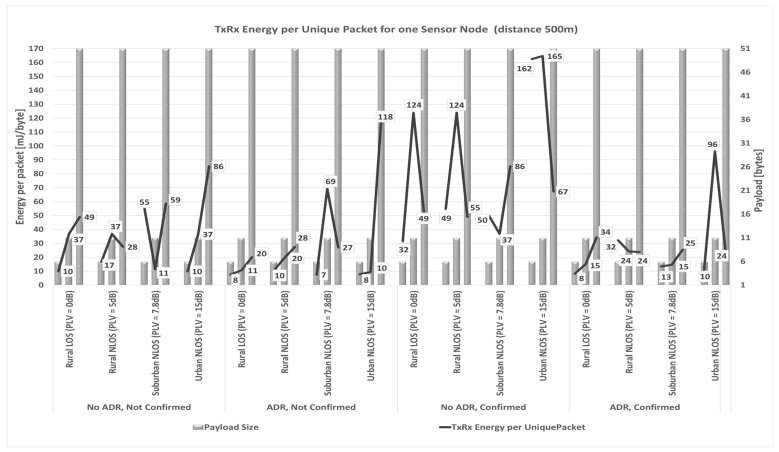
TxRx energy consumption per unique packet of one sensor node.

According to the results presented in Figure 6, it can be observed that the substantial energy in the transmit (Tx) and receive (Rx) states can be reduced by disabling confirmed messages. In addition, since analyzing energy consumption optimization regarding the reduction in acknowledged transmissions, the impact of the duty cycle limit on the downlink capabilities of the gateway is also considered. The gateway cannot acknowledge all confirmed messages if only the default channels are utilized. Consequently, the number of retransmitted packages increases. Regarding the collected results for the deployed LoRaWAN sensor network scenarios, it can be concluded that the number of acknowledged frames must be minimized as much as possible to avoid capacity and energy drain.

The communication channel variance, measured in dB, concerning the average path loss, i.e., path loss variance (PLV), is a consequence of varying propagation characteristics of the signal in space and time. As presented, the channel dynamics significantly impact the performance of a LoRaWAN network and the energy efficiency of the nodes.

In general, the following can be stated. First, the ADR mechanism attempts to achieve a robust signal while considering energy consumption. This is achieved by lowering the data rate when the channel conditions worsen and increasing the data rate when the SNR conditions improve.

**Figure 7:**
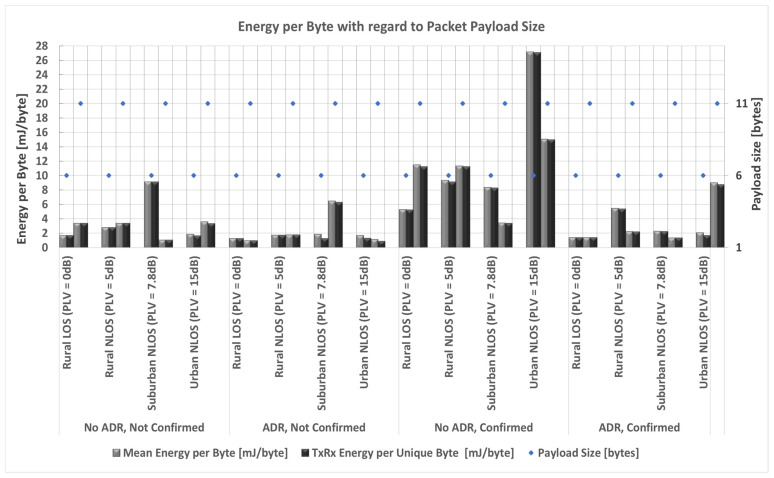
Consumed energy per byte for different packet payload sizes.

According to the results presented in Figure 7, it is evident that ADR should be used in the analyzed scenarios since substantial energy savings can be achieved by enabling ADR. ADR reduces the energy consumption of the sensor nodes. First, it optimizes the LoRa parameters in such a way that nodes do not transmit with more power than needed. Secondly, this inherently diverges the utilized spreading factors, yielding fewer collisions. This demonstrates the importance of including ADR in assessing and optimizing transmission parameters in LPWANs to ensure a long battery life of sensor nodes.

However, ADR reacts slowly in situations with many devices or when a significant channel variance is present. The current ADR mechanism is unable to adjust for rapid channel variances. This effect is negligible if confirmed messages (i.e., retransmissions) are employed to ensure receiving each intended message. If no acknowledgments are used, the data extraction rate decreases when the channel variance increases.

The nodes are unable to adapt adequately to the channel. Concerning the data extraction rate, it is better not to enable an adaptive data rate when not using confirmed messages. However, this results in higher energy consumption.

Despite the beneficial effects of increasing the payload size, sending more bytes per packet increases the total number of bytes sent redundantly. After receiving 20 uplink messages, the network will respond with adequate ADR parameters to accommodate non-optimal propagation [88]. For larger payload sizes, this implies that more bytes have been sent before the LoRa parameters are adjusted to the channel. In addition, ADR changes the parameters in steps, which causes an even slower adaption to the propagation environment for larger payload sizes. This effect is notable when observing the energy consumption of nodes with a slow data transmission rate. This results in higher energy consumption. Therefore, LoRa devices should send smaller packets to adapt to the channel faster since this will reduce air time and energy consumption for packets sent with non-optimal parameters.

The energy per useful bit is a function of the maximum range and different payload sizes. With high SF values and an increase in the payload size, the energy per useful bit decreases. Also, with low SF values, the payload variation does not substantially affect energy per bit. 

In extension to these analyses, the analyses carried out as part of this work consider fixed, predefined payload sizes when estimating the transmission rate values. Therefore, the corresponding transmission rate calculated is used for each scenario related to the estimation of energy consumption based on the selected packet size. The conclusions based on the obtained results fulfill those obtained in [3,4,15], and indicate that, regardless of SF values, if the payload size decreases, the energy per useful bit also decreases, as well in the chosen scenarios of optimal packet sizes in bytes for the given case study.

**Figure 8:**
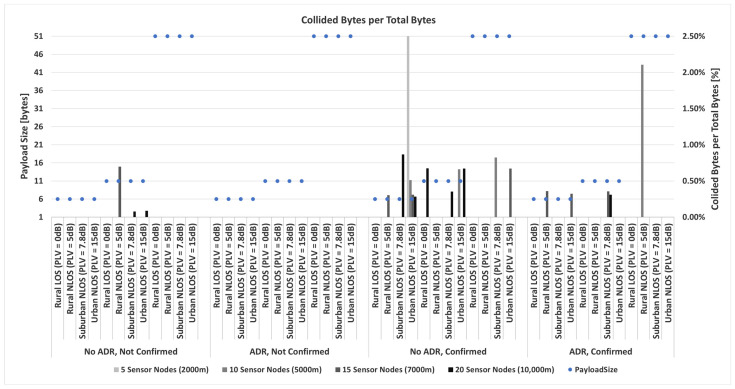
Percentage of bytes that collided out of the total number of bytes sent.

Delivering a large number of packets in a short time by a more significant number of nodes causes bursts of collisions, as presented in Figure 8.

In LoRaWAN, the reliability of packet delivery is achieved through the acknowledgment of packets in the downlink. However, sending confirmation increases the energy consumption in the network. Therefore, when there is a certain tolerance for packet loss, as is the case when sending not so time-critical data, for instance, data related to the state of the environmental and agronomic parameters, it is possible to omit sending confirmations. This is especially appropriate in cases of implementing sensor networks with a smaller number of nodes and sending data less often, where the frequency of possible collisions is reduced. Moreover, this is also appropriate in cases where high signal quality is ensured and there are no significant losses during packet transmissions.

Considering what has been stated, it can be concluded that in some cases, acknowledgments of packet delivery are not sent due to savings in energy consumption. However, to ensure the successful delivery of the amount of data needed to gain insight into the value of environmental and agronomic data necessary for crop condition assessment, it is recommended to use packet sequence numbers nevertheless. In this way, the server could check the received traffic, and in the critical case of a more considerable difference between the sequential numbers of the two successively received packets, it can inform the sensor node that ADR should be applied and continue sending data according to its recommendations.

The LoRa network server can monitor the detailed radio information from all gateways that receive a given frame. These data include information about the gateway, extended unique identifier (EUI) of the networking devices (sensor node), server timestamp, acknowledgment flag as set by the device, frame counter (FCnt), and encrypted data payload. In addition, some extended radio information such as the radio frequency, spreading factor, bandwidth, coding rate, the gateway’s Rx UTC and GPS time, and the gateway’s position (latitude and longitude) can also be added. According to this information, the network server can control the sequence number of the particular packet received from the selected sensor node.

A standard comma-separated value (.CSV) file which is presented to the receiving end contains the following columns:
EUI, timestamp, FCnt, frequency, data rate, RSSI, SNR, gateway EUI, port, data

E.g.,:
0004A30B00FFEF62,1655557243123,161,868500000,SF11 BW125 4/5,-115,-3.5,024B0BFFFF0310B2,1,693e0001bf3eb0020000ff 0004A30B00FFEF62,1655557843123,162,867500000,SF11 BW125 4/5,-102,-12.8,024B0BFFFF0310B2,1,693e4001bf3e98020000ff 0004A30B00FFEF62,1655558443123,163,867700000,SF11 BW125 4/5,-115,-2.5,024B0BFFFF0310B2,1,693e4001bf3e80020000ff 0004A30B00FFEF62,1655559043103,164,868100000,SF11 BW125 4/5,-118,-2,024B0BFFFF0310B2,1,693e4001bd3e70020000ff 0004A30B00FFEF62,1655560243103,166,868300000,SF11 BW125 4/5,-115,-7.8,024B0BFFFF0310B2,1,693e8001bd3e58000000ff ⋯

Therefore, it is evident that, based on continuous monitoring of the counter (FCnt), an insight into its changes can be gained to define an arbitrary maximum deviation from two consecutive counter values before the application of ADR is requested.

In the case that the application server contains some data, for instance, about ADR usage, which it needs to transmit to a specific sleeping sensor that sends data periodically, when that sensor is a Class A device, the application server must wait for an uplink from the sensor before it can send its data [72]. The application server immediately transmits the downlink once the uplink is received. Upon receipt of the downlink, the sensor goes back to sleep. This way, even if some overhead is present, energy savings can be achieved.

**Figure 9:**
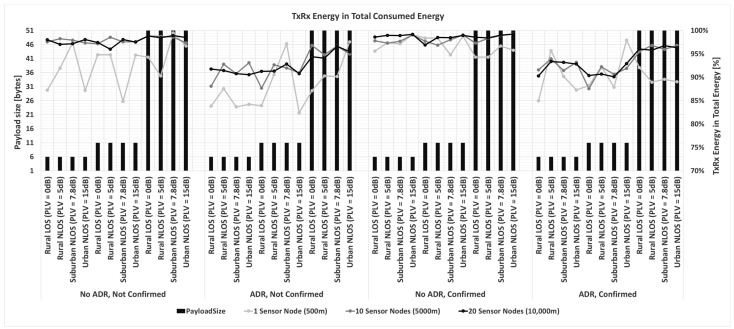
Percentage of TxRx energy in the total consumed energy.

**Figure 10:**
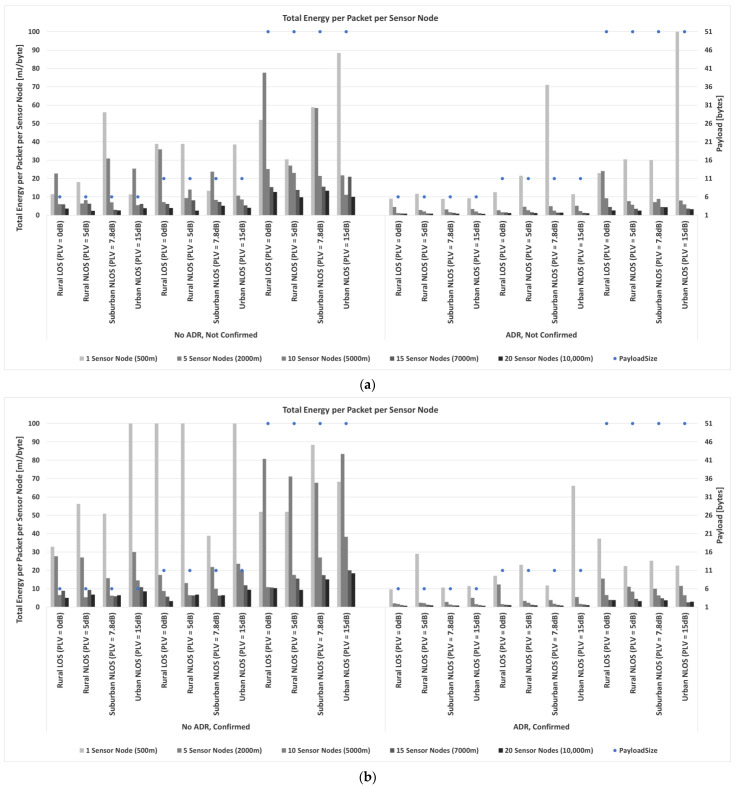
Total consumed energy per packet per sensor node. (**a**) Unconfirmed packets with and without ADR; (**b**) Confirmed packets with and without ADR.

Furthermore, as can be concluded from the collected results presented in Figure 9, the transmit (Tx) and receive (Rx) state energies are the most significant contributors to total energy consumption. However, the sleep mode energy has a more significant share in the total energy if ADR is used while the confirmations are omitted, as presented in Figure 10. The results show that optimizing the LoRa parameters such as SF, CR, and payload size is a crucial element in reducing the consumed energy by the sensor node. The total consumed energy per useful bit is a function of the payload at different spreading factors (SF). For the more excellent value of SF, more time is taken to send a packet, so more consumed energy is needed to transmit data. Moreover, when the coding rate (CR) decreases, the time on air and, consequently, the consumed energy increases. Furthermore, there is a trade-off between the LoRaWAN communication range, the spreading factor, and the transmission power. The range is a function of SF at different transmission powers. If the SF increases, the LoRaWAN range increases as well. Moreover, the LoRaWAN range increases with increasing transmission power. It can be concluded that the theoretical maximum range that can be achieved at the determined power level is obtained with the highest SF.

Saving electricity is one of the fundamental priorities in all aspects of modern life [89,90,91]. The results of this work show the saving of electricity in precision agriculture. Limitation of the proposed study relate to the fact that only data for three carefully selected sizes of packets that contain data essential for precision agriculture are presented in the paper. However, these sizes correspond to one of the smaller, average and the largest packet that can be sent over the network. Moreover, it was shown that a 6-bit packet is sufficient for transferring the same amount of data as an 11-bit packet.

## 5. Conclusions and Recommendation

This paper analyzed the energy-efficient operation of a LoRa-based wireless sensor network intended for precision agriculture. This type of sensor network may be used for crop condition monitoring by measuring essential environmental parameters. The paper proposed a concise presentation of information in the packet payload (a 6-byte payload format, which provides the same amount of useful information as the 11-byte payload used with commercially available devices). The given example of the selected smaller packet size is a more suitable solution in terms of energy saving.

Furthermore, the difference in the analyses carried out in this paper compared to the analyses carried out in the literature so far lies in the definition of an adequate transmission rate. While the analyses carried out so far have assumed fixed values of the transmission rate, in this paper it was assumed that the transmission rate changes with each sending, considering the amount of data in the sent packets, i.e., the size of the packet. This affects the results, because in this paper it was proven that for better energy efficiency of communication, it is important to adjust the transmission speed to the actual size of the packet. So, the high transmission rate can be more energy-efficient method when sending smaller packets.

By testing LoRa communication modules in real case study conditions, the results collected through field measurements, simulations, and laboratory tests were compared. An optimized energy model for LoRa sensor nodes was presented. It was shown that the consumed energy changes with different parameter settings. Optimizing LoRa parameters, such as SF and CR, regarding the required long-range communication is crucial in reducing the energy consumed by the sensor nodes in the case study areas. However, it is proven that, regardless of SF values, if the payload size decreases, the energy per useful bit decreases as well.

Moreover, it is evident that ADR should be used in the analyzed scenarios since substantial energy savings can be achieved by enabling ADR. After receiving 20 up-link messages, the network will respond with adequate ADR parameters to accommodate non-optimal propagation. Therefore, LoRa devices should send smaller packets to adapt to the channel faster since this will reduce air time and energy consumption for packets sent with non-optimal parameters.

Finally, it was concluded that receiving a transmission acknowledgment consumes energy, which considerably reduces the sensor node lifetime. Therefore, another contribution of the paper is the graph showing the limits of reliable data transmission. According to the obtained graph, it is possible to identify areas within which it is not necessary to send acknowledgments, which can significantly reduce energy consumption based on the last measured SNR and RSSI values. Moreover, the selection of the frequency of sending acknowledgments must also be compliant with the ETSI and LoRaWAN standards, which also apply to gateways.

## Figures and Tables

**Table 1:** Transceiver current consumption.

Transceiver	Current Consumption							
	Transmit					Receive	Sleep	References
	20 dBm	14 dBm	13 dBm	7 dBm	2 dBm			
HopeRF RFM95/96/97/98(W)	120 mA	-	29 mA	20 mA	-	11.5 mA (min. 10.8 mA, max. 12.1 mA)	0.2 µA (max. 1 µA)	[54]
HopeRF HM-TRLR-LF/HFS	120 mA	-	35 mA	-	-	16 mA (min. 15 mA, max. 18 mA)	2 µA (max. 3 µA)	[59]
	133 mA	-	-	-	-	16.3 mA	7.7 µA	[60]
Semtech SX1276	120 mA	-	-	20 mA	-	11.5 mA (min. 10.8 mA, max. 12.0 mA)	0.2 µA (max. 1 µA)	[61]
	-	-	-	-	-	14 mA	0.17 mA	[34]
	-	-	-	-	-	16.6. mA	3.7 mA	[35,62]
Semtech SX1272	124 mA	-	-	18 mA	-	10.5 mA or 11.2 mA	0.1 µA (max. 1 µA)	[63]
	-	-	-	-	-	11 mA	2 µA	[31,64]
	-	-	-	-	26 mA	12 mA	40 µA	[32,64]
	-	-	-	-	-	20 mA	70 µA	[33,65]
Microchirp RN2482	-	38.9 mA	-	-	-	14.2 mA	up to 100–150 µA	[58,60,66,67]
	-	48 mA	-	-	-	17.2 mA	3.4 mA	[62,66]
	-	38.5 mA	-	-	23.9 mA	-	-	[68]
	-	-	-	-	-	46 mA	34 mA	[69]

**Table 2:** Transceiver power consumption.

Transmit Power for the Defined Finite Transmit Power States						
Transceiver	Transmit Mode	RFOP = +7 dBm, on RFO_LF/HF Pin	RFOP = +13 dBm, on RFO_LF/HF Pin	RFOP = +17 dBm, on PA_BOOST	RFOP = +20 dBm, on PA_BOOST	Reference
SX1272	Power consumption (mW)	95.4	95.4	297	412	[15]
RFM95/96/97/98(W)		66	95.7	287	396	[54]

**Table 3:** Energy profiles of the sensor nodes.

Energy Profile Used in the Analyses						
Sensor State	Power [54]					Duration (ms) [70,71,73]
Sleep	4.95 × 10^−3^ mW					-
Processing	19.14 mW					5 ms
Tx prep.	12.5 mW					40 ms
Tx	2 dBm	7 dBm	14 dBm	17 dBm	20 dBm	Equation in [3]
	25 mW	66 mW	125 mW	287 mW	396 mW	
Wait Rx1	4.95 × 10^−3^ mW					1 × 10^3^ ms
Wait Rx2	4.95 × 10^−3^ mW					1 × 10^3^–len (state Rx1)
Rx prep.	5.94 mW					3.4
Rx1	37.95 mW					air_time (DR = DR_tx)
Rx2	35.64 mW					air_time (DR = 3)
Rx post proc.	5.94 mW					10.7

**Table 4:** Packet payload of 11 bytes.

Packet Payload Format: 11-Byte Payload											
Measured Parameter	Type	Battery	Temperature	T_min	T_max	Humidity	Atmospheric Pressure	Irradiation	Max Irradiation	Rain	Min Time between Rain Gauge Clicks
Bit start position	1st	3rd bit	8th bit	19th bit	25th bit	31st bit	40th bit	54th bit	64th bit	73rd bit	81st bit
No. of bits	2	5	11	6	6	9	14	10	9	8	8
Value in binary	01	10100	10011111000	000000	000000	011011111	10011111010110	0000000001	000000000	00000000	11111111
Value in units	1	4	27.2	0	0	44.6	100,990	2	0	0	255
Units	N/A	V	°C	°C	°C	%	Pa	W/m^2^	W/m^2^	Pulses	Seconds
Resolution	1	0.05	0.1	0.1	0.1	0.2	5	2	2	1	1
Max no. of values	4	32	2048	64	64	512	16,384	1024	512	256	256
Min–max value	0–3	3–4.55	−100–104.7	0–6.3	0–6.3	0–102.2	50,000–131,920	0–2046	0–1022	0–255	0–255
Req min–max values	0–3	3–4.5	−50–80	0–3	0–3	0–100	60,000–128,000	0–1500	0–100	0–25	1–255
Check	OK	OK	OK	OK	OK	OK	OK	OK	OK	OK	OK

**Table 5:** Packet payload of 6 bytes.

Packet Payload Format: 6-Byte Payload						
Measured Parameter	Battery	Temperature	Humidity	Atmospheric Pressure	Irradiation	Rain
Bit start position	1st bit	6th bit	14th bit	23rd bit	35th bit	44th bit
No. of bits	5	8	9	12	9	5
Value in binary	10100	10000110	011011111	100101101011	000000001	00000000
Value in units	4	27.0	44.6	100,987	3	0
Units	V	°C	%	Pa	W/m^2^	Pulses
Resolution	0.05	0.5	0.2	17	3	1
Max no. of values	32	256	512	4096	512	32
Min–max value	3–4.55	−40–87.5	0–102.2	60,000–129,632	0–1536	0–32
Req min–max values	3–4.5	−50–80	0–100	60,000–128,000	0–1500	0–25
Check	OK	OK	OK	OK	OK	OK

**Table 6:** Maximum number of packets according to duty cycle.

Output	SF6	SF7	SF8	SF9	SF10	SF11	SF12
SF	6	7	8	9	10	11	12
DE	0	0	0	0	0	1	1
Tsym (ms)	0.5	1.0	2.0	4.1	8.2	16.4	32.8
Tpreamble (ms)	6.3	12.5	25.1	50.2	100.4	200.7	401.4
Application payload size	51 bytes						
payloadSymbNb (symbols)	123	103	93	83	73	83	73
Tpayload (ms)	63.0	105.5	190.5	340.0	598.0	1359.9	2392.1
Tpacket (ms)	69.2	118.0	215.6	390.1	698.4	1560.6	2793.5
TTN Fair Access Policy (messages/day)		254	139	76	42	19	10
TTN Fair Access Policy [messages/hour]		10.6	5.8	3.2	1.8	0.8	0.4
Duty cycle (s): 0.1%	69.2	118.0	215.6	390.1	698.4	1560.6	2793.5
Duty cycle (s): 1%	6.9	11.8	21.6	39.0	69.8	156.1	279.3
Duty cycle (s): 10%	0.7	1.2	2.2	3.9	7.0	15.6	27.9
Application payload size	11 bytes						
payloadSymbNb (symbols)	53	48	43	38	33	38	33
Tpayload (ms)	27.1	49.2	88.1	155.6	270.3	622.6	1081.3
Tpacket (ms)	33.4	61.7	113.2	205.8	370.7	823.3	1482.8
TTN Fair Access Policy (messages/day)		486	265	145	80	36	20
TTN Fair Access Policy (messages/hour)		20.3	11.0	6.1	3.4	1.5	0.8
Duty cycle (s): 0.1%	33.4	61.7	113.2	205.8	370.7	823.3	1482.8
Duty cycle (s): 1%	3.3	6.2	11.3	20.6	37.1	82.3	148.3
Duty cycle (s): 10%	0.3	0.6	1.1	2.1	3.7	8.2	14.8
Application payload size	6 bytes						
payloadSymbNb (symbols)	48	38	38	33	28	33	28
Tpayload (ms)	24.6	38.9	77.8	135.2	229.4	540.7	917.5
Tpacket (ms)	30.8	51.5	102.9	185.3	329.7	741.4	1318.9
TTN Fair Access Policy (messages/day)		583	291	161	90	40	22
TTN Fair Access Policy (messages/hour)		24.3	12.1	6.7	3.8	1.7	0.9
Duty cycle (s): 0.1%	30.8	51.5	102.9	185.3	329.7	741.4	1318.9
Duty cycle (s): 1%	3.1	5.1	10.3	18.5	33.0	74.1	131.9
Duty cycle (s): 10%	0.3	0.5	1.0	1.9	3.3	7.4	13.2

**Table 7:** Battery lifespan.

Application Payload (Bytes)	Messages per Hour	Configuration	Periodicity T_off_ (min:s)	Battery TTL (years/months/weeks)	Battery Type				
					AAA (Alkaline, 800 mAh)	Li-Ion (260 mAh)	AA (Alkaline, 2500 mAh)	Li-Ion (1000 mAh)	Li-Ion (2000 mAh)
51	12	SF12/125 kHz	4:57	Worst case	1 m	1 m	3 m	4 m	7 m 3 w
		SF7/125 kHz	5:00	Best case	9 m	9 m	2 y 4 m 1 w	2 y 10 m 3 w	5 y 9 m 2 w
	6	SF12/125 kHz	9:57	Worst case	2 m	2 m	6 m 1 w	7 m 3 w	1 y 3 m 3 w
		SF7/125 kHz	10:00	Best case	1 y 4 m 3 w	1 y 5 m	4 y 4 m 3 w	5 y 5 m	10 y 10 m 1 w
11	12	SF12/125 kHz	4:57	Worst case	2 m	2 m	6 m 1 w	7 m 3 w	1 y 3 m 3 w
		SF7/125 kHz	5:00	Best case	10 m 1 w	10 m 1 w	2 y 8 m 1 w	3 y 4 m	6 y 8 m
	6	SF12/125 kHz	9:57	Worst case	4 m	4 m	1 y 3 w	1 y 3 m 2 w	2 y 7 m 1 w
		SF7/125 kHz	10:00	Best case	1 y 7 m 1 w	1 y 7 m 1 w	5 y	6 y 2 m	12 y 4 m 1 w
6	12	SF12/125 kHz	4:57	Worst case	2 m 1 w	2 m 1 w	7 m 1 w	9 m	1 y 6 m
		SF7/125 kHz	5:00	Best case	10 m 2 w	10 m 2 w	2 y 8 m 3 w	3 y 4 m 2 w	6 y 9 m
	6	SF12/125 kHz	9:57	Worst case	4 m 2 w	4 m 2 w	1 y 2 m 2 w	1 y 5 m 3 w	2 y 11 m 3 w
		SF7/125 kHz	10:00	Best case	1 y 7 m 2 w	1 y 7 m 2 w	5 y 3 w	6 y 3 m	12 y 6 m

**Table 8:** Measured packet delivery ratio.

Packet (P) Size:	Pmax								Pmin							
Transceiver Power Mode:	Low Power (14 dBm)				High Power (20 dBm)				Low Power (14 dBm)				High Power (20 dBm)			
SF:	7		10		7		10		7		10		7		10	
CR:	4/5	4/8	4/5	4/8	4/5	4/8	4/5	4/8	4/5	4/8	4/5	4/8	4/5	4/8	4/5	4/8
Scenario:	Delivered Packets (DP) in Relation to Sent Packets:															
**Close Proximity (distance: <100 m)**	100%	100%	100%	100%	100%	100%	100%	100%	100%	100%	100%	100%	100%	100%	100%	100%
**Urban NLOS (distance: 500 m)**	100%	100%	100%	100%	100%	100%	100%	100%	100%	100%	100%	100%	100%	100%	100%	100%
**Urban NLOS (distance: 2000 m)**	0%	0%	40%	100%	40%	40%	40%	100%	40%	40%	60%	100%	60%	60%	60%	100%
**Suburban NLOS (distance: 5000 m)**	0%	0%	60%	60%	40%	0%	40%	60%	40%	40%	60%	80%	40%	80%	40%	80%
**Rural LOS (distance: 7000 m)**	40%	60%	80%	100%	80%	100%	60%	100%	40%	80%	80%	100%	80%	80%	60%	100%
**Rural NLOS (distance: 7000 m)**	0%	0%	40%	0%	0%	0%	0%	0%	0%	0%	40%	0%	0%	0%	0%	60%

**Table 9:** Packet payloads.

Packet Payload in Bytes	Maximal Total Packet Size in Bytes	Transmission Rate per Hour
6	14	0.19 × 10^−3^
11	19	0.25 × 10^−3^
51	59	0.79 × 10^−3^

## Data Availability

The data presented in this study are openly available in repository FigShare at 10.6084/m9.figshare.23663982.
